# Delay in Diagnosis of Duchenne Muscular Dystrophy

**DOI:** 10.15844/pedneurbriefs-29-1-4

**Published:** 2015-01

**Authors:** Vamshi K. Rao, Nancy L. Kuntz

**Affiliations:** 1 Division of Neurology, Department of Pediatrics, University of Nebraska Medical Center, Omaha, NE; 2 Division of Neurology, Ann & Robert H. Lurie Children's Hospital of Chicago, Chicago, IL; 3 Departments of Pediatrics and Neurology, Northwestern University Feinberg School of Medicine, Chicago, IL

**Keywords:** Muscular Dystrophy, Developmental Delay, Diagnosis

## Abstract

Investigators from Johns Hopkins Hospital, Baltimore, MD, retrospectively reviewed 179 records of patients with Duchenne muscular dystrophy (DMD) evaluated between 1989 and 2012. Diagnosis was confirmed by genetic testing or muscle biopsy, and clinical data were complete in 107 patients.

Investigators from Johns Hopkins Hospital, Baltimore, MD, retrospectively reviewed 179 records of patients with Duchenne muscular dystrophy (DMD) evaluated between 1989 and 2012. Diagnosis was confirmed by genetic testing or muscle biopsy, and clinical data were complete in 107 patients. Cognitive delay (special education placement or delay in grade progression during primary school) was present in 45%, and a delay in walking (after the 16^th^ month) in 42%. Cognitive delay and delay in walking were strongly associated (P<.0001). The severity of the motor phenotype assessed by age of lost independent ambulation was not associated with delay in walking or cognitive impairment. The mean age of lost independent ambulation was 10.9 years. The age of diagnosis for this cohort was not related to delay in walking or cognitive status. The mean age of diagnosis was 5.1 +/- 2 years. Neither the age of lost independent ambulation nor the age of diagnosis was associated with age of walking. A subject with DMD who walked at or after 16 months of age had three times the risk of also progressing more slowly in school. DMD should be considered in the differential diagnosis of global developmental delay. Age of walking is influenced more by cognitive delay than by motor phenotype. Earlier diagnosis may be possible. Serum creatine kinase (CK) should be considered for any preschool boy who begins to walk later than 16 months of age. [1]

COMMENTARY. DMD is a disorder of progressive muscle weakness due to a mutation in the dystrophin gene leading to an absence of the dystrophin protein. In the muscle, dystrophin protein is a part of a complex that anchors the muscle cytoskeleton to the extracellular framework that resists mechanical stress thereby preventing injury. In the brain, possibly a signaling role predominates with presence of the full length dystrophin isoforms in the GABAergic synapses in the cortex, hippocampus and cerebellum [2] and short isoforms localizing to the glia. Impaired full scale, verbal and performance intelligence quotients have been noted in large cohorts of DMD patients [3] along with comorbid psychiatric disorders such as ADHD (11-20%), Autism spectrum disorder (3-4%) and OCD (5-60%) [4].

Mutations affecting short isoforms contribute to more cognitive delay than full-length isoforms where only a minimal frequency of intellectual impairment is noted [5]. Determining factor for cognitive abnormalities in DMD seems to be a differential cumulative loss of brain expressed isoforms determined by mutation patterns in the gene that seem to localize to a yet unknown glial role of brain dystrophin.

There is ample evidence to suggest that the presence of dystrophin in the skeletal muscle and brain in DMD is associated with delay in walking and cognitive impairments. There are ongoing studies to ascertain a specific genotype-phenotype correlation for the known mutations with respect to walking and cognitive delays. In the interim, until newborn screening is in place for DMD, a simple laboratory test such as CK for all children with delayed walking (at least >16 months) and cognitive impairment will lead to early diagnosis, symptom management and with currently promising clinical trials, a treatment.
